# Compounding Parenteral Products in Pediatric Wards—Effect of Environment and Aseptic Technique on Product Sterility

**DOI:** 10.3390/healthcare9081025

**Published:** 2021-08-10

**Authors:** Sonja Virtanen, Karmen Kapp, Maria Rautamo, Lotta Schepel, Carita Lindén-Lahti, Cristina D. Cruz, Päivi Tammela

**Affiliations:** 1 Drug Research Program, Division of Pharmaceutical Biosciences, Faculty of Pharmacy, P.O.Box 56 (Viikinkaari 5E), University of Helsinki, FI-00014 Helsinki, Finland; sonja.a.virtanen@outlook.com (S.V.); karmen.kapp@helsinki.fi (K.K.); cristina.durantecruz@helsinki.fi (C.D.C.); 2 HUS Pharmacy, HUS Helsinki University Hospital and University of Helsinki, Stenbäckinkatu 9B, 00290 Helsinki, Finland; maria.rautamo@hus.fi (M.R.); lotta.schepel@hus.fi (L.S.); carita.linden-lahti@hus.fi (C.L.-L.)

**Keywords:** parenteral products, compounding, aseptic technique, sterility test, pediatrics, patient safety

## Abstract

Parenteral products must be compounded using an aseptic technique to ensure sterility of the medicine. We compared the effect of three clinical environments as compounding areas as well as different aseptic techniques on the sterility of the compounded parenteral product. Clinical pharmacists and pediatric nurses compounded 220 samples in total in three clinical environments: a patient room, a medicine room and biological safety cabinet. The study combined four methods: observation, environmental monitoring (settle plates), monitoring of personnel (finger dab plates) and sterility testing (membrane filtration). Of the compounded samples, 99% were sterile and no significant differences emerged between the clinical environments. Based on the settle plates, the biological safety cabinet was the only area that fulfilled the requirements for eliminating microbial contamination. Most of the steps on the observation form for aseptic techniques were followed. All participants disinfected their hands, wore gloves and disinfected the septum of the vial. Non-contaminated finger dab plates were mostly detected after compounding in the biological safety cabinet. Aseptic techniques were followed relatively well in all environments. However, these results emphasize the importance of good aseptic techniques and support the recommendation of compounding parenteral products in biological safety cabinets in clinical environments.

## 1. Introduction

Parenteral products are sterile products that are administered as an injection, infusion or implantation [1]. These must be manufactured and compounded using materials and methods (aseptic techniques) that ensure sterility of the product. Administration of a contaminated parenteral product can cause the patient significant harm, including bloodstream infections, sepsis, meningitis and death [2,3,4,5,6]. Pediatric patients are particularly vulnerable to contaminated parenteral products due to their undeveloped immune systems [7]. 

Errors in aseptic techniques might lead to contamination of the compounded product; however, there are large variations in reported error rates (4.0–98.7%) of aseptic techniques [8,9,10,11]. The most common errors in aseptic techniques are insufficient hand hygiene and cleaning of drug ampoules, vials and the compounding area. Adoption of aseptic techniques is the most efficient way to prevent contamination of sterile products [12,13,14]. Microbial contamination of compounded products is more likely to occur when compounding is done in a clinical setting than in a pharmaceutical environment [15,16]. High amounts of airborne microbials in the environment as well as compounding products as individual batches increase the risk of contamination [11,13,14,15,16]. Lower contamination rates occur when a more experienced person performs the compounding [13,17].

Most parenteral products must be compounded (e.g., dissolved or diluted) before administration. According to the Finnish Medicines Agency (Fimea), compounding of sterile products should be done in a hospital pharmacy whenever possible [18]. Fimea further states that a biological safety cabinet (BSC) should be the primary compounding environment for parenteral products in hospital wards or other health care units [18]. 

Previous studies have focused on comparing the contamination rates of products compounded in clinical or pharmaceutical environments [13,14,15,16]. To the knowledge of the authors, different environments used for compounding in hospital wards have not been investigated previously, and thus, the contamination risk related to compounding of parenteral products in health care units is unknown.

The aim of this study was to compare the effect of three environments for compounding of parenteral products in pediatric wards, namely, the patient’s room (PR), medicine room (MR) and biological safety cabinet (BSC), on the contamination rate of the compounded product. Another objective was to determine how well aseptic techniques are followed during compounding of parenteral products in hospital wards.

## 2. Materials and Methods

### 2.1. Study Design and Participants

The study was conducted in two separate pediatric wards at HUS Helsinki University Hospital between March and May 2019. One of the wards provides intensive care and the other emergency care. Altogether 12 volunteers, one clinical pharmacist and five nurses from each ward were recruited to the study by nurse managers or clinical pharmacists. All participants were experienced in medicine compounding. 

Three environments were used for compounding in the wards: PR, MR and BSC. In the PR, a separate cart or small desk served as the compounding area. Compounding was done in the empty PR to prevent any disturbance to patients or their medical care. In the MR, compounding was performed on a desk, and other nurses could be present in the room simultaneously. There is no significant difference in the air flow between the PR and MR. The BSC was situated either in the MR or in a separate room with an entrance from the MR. The following BSCs were used: Kojair SL-66 Silver, 2009 (ward 1) and BioWizard Silver SL-170 C Blue Series, 2016 (ward 2). Ward 2 was recently renovated, hence the BSC is newer in ward 2.

### 2.2. Compounding of Parenteral Product Samples

The compounding of test samples was designed to simulate the procedure of dissolving, diluting and dispensing commonly used cefuroxime for intravenous infusion. The test samples were prepared by adding 5 mL of sterile water for injections (Braun, Melsungen, Germany) to a vial containing sterile sodium chloride (NaCl) powder (HUS Pharmacy, simulating cefuroxime powder). After dissolving NaCl, the solution was drawn into two syringes (BD Plastipak, Mendig, Germany), 2 mL in each, where it was further diluted to 10 mL with sterile 0.9% NaCl solution (Braun, Melsungen, Germany). Detailed instructions for the compounding are provided in Supplementary Materials—Document S1.

**Figure 1:**
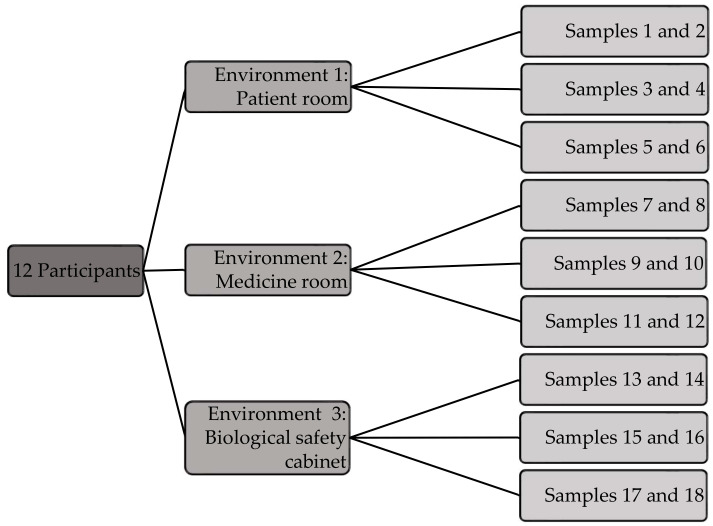
Compounding of parenteral product samples (N = 220). One participant compounded four extra samples to evaluate the variation in sterility within one batch.

A total of 220 samples were collected during the study (Figure 1). Each participant compounded 18 samples, 6 in each of the aforementioned environments. Samples were compounded on three separate days, six samples at a time. One participant compounded four extra samples in the PR to evaluate the variation in sterility within one batch (Figure 1).

### 2.3. Study Methods

**Figure 2:**
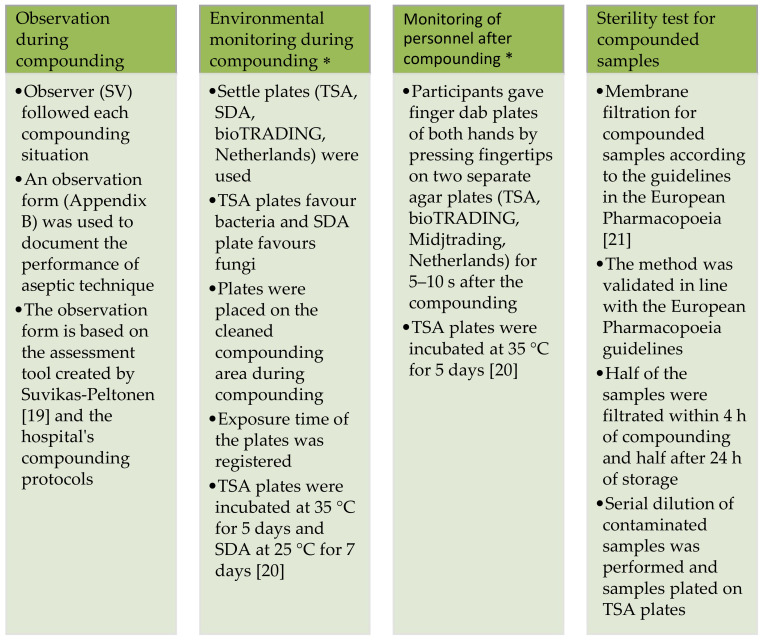
Study methods of observation, environmental monitoring, monitoring of personnel and sterility testing of product samples were used to evaluate the effect of aseptic technique and environment on product sterility. TSA = tryptic soy agar, SDA = sabouraud dextrose agar. * European Union Good Manufacturing Practices (EU GMP) guidelines for recommended limits for microbiological monitoring of clean areas during operation were applied in the monitoring of environment and personnel [19,20,21].

The study combined four different methods: observation, environmental monitoring, monitoring of personnel and sterility testing (Figure 2).

### 2.4. Sterility Test and Identification of Contaminants

Membrane filtration was used as a sterility test for the compounded samples. One duplicate was filtrated within 4 h of compounding to simulate a situation where the product is administered immediately after compounding. The second duplicate was filtrated after 24 hours’ storage to simulate a situation where the compounded product is stored in a refrigerator for 24 h in the wards prior to administration. The samples were stored at 4 °C before membrane filtration. Membrane filtration was performed in an aseptic laboratory in a laminar flow hood cabinet using a Sentino^®^ Microbiology Pump (Pall Corporation, Port Washington, NY, USA) and MicroFunnel™ filters (Pall Corporation, Port Washington, NY, USA) with a membrane pore size of 0.2 µm. After filtration, the membrane was cut into two halves. One half was transferred into a thioglycolate broth (Neogen Culture Media, Heywood, UK) and the other half into a tryptic soy broth (TSB, Neogen Culture Media, Heywood, UK). Both media were incubated for 14 days, TSB at 25 °C and thioglycolate broth at 35 °C, after which they were visually examined for turbidity. If turbidity was detected, 100 µL of five serial dilutions of the media were plated on a tryptic soy agar (TSA) plate (Neogen Culture Media, Heywood, UK) and incubated at 35 °C for 5 days. The plate was visually examined after incubation and the number of colony-forming units (CFUs) was calculated. A stock solution of each phenotypically different colony was prepared in order to identify the contaminants using an OmniLog ID System (Biolog, Hayward, CA, USA). The stock solutions were stored at −80 °C prior to analyses with the OmniLog ID System [22].

### 2.5. Analysis of Data

Microsoft^®^ Excel was used to collect and analyze the data. IBM^®^ SPSS^®^ Statistics 25 (IBM, Armonk, NY, USA) was used for statistical analyses (ANOVA). Observations were collected from the structured observation forms (Supplementary Materials—Document S2), and frequencies and means were determined.

### 2.6. Study Ethics

A research permit was granted by HUS. Ethics approval was not needed because the study does not include patient data. All participants received written information about the study before providing written informed consent. Participants were also informed about the option to withdraw from the study at any time.

## 3. Results

### 3.1. Observation during Compunding

The observations revealed that most steps on the observation form for aseptic techniques were followed; however, some deviations from the hospital guidelines were noted (Table 1). Only one participant completed every step during one of the compounding situations in PR. There was at least one deficiency in aseptic technique in all other compounding situations. All participants wore non-sterile gloves during compounding and disinfected their hands before putting on the gloves. However, inadequate use of the protective gown was common. Deficiencies were also found in the cleaning and disinfection processes, regarding both the compounding area and the materials and products used during the compounding. The septum of the vial was disinfected before piercing it the first time; however, if the septum was pierced a second time the disinfection was insufficient. The BSC was used mainly according to the protocol. The majority (92%) kept the airflow of the BSC on for 15 min before starting the compounding. Usage of a sterile drape cover on the benchtop during compounding in the BSC and cleaning the cabinet after compounding was done in 50% of the situations. None of the participants used a surgical face mask or a hair cover while compounding in the BSC.

### 3.2. Environmental Monitoring during Compounding

Monitoring of the environment shows that the BSC is a less contaminating setting for compounding than the MR or PR based on the microbial growth on settle plates from these environments (*p* < 0.001, Table 2). Despite differences between the two wards regarding the means of CFUs on the settle plates, these differences were not statistically significant. Personal variations in the number of CFUs on the settle plates were seen between different compounding situations in the same surroundings, especially during compounding in the MR and PR. Furthermore, the results reveal that the number of non-contaminated settle plates is fairly high in all three environments, namely 97%, 71% and 61% for BSC, MR and PR, respectively.

Comparison of the settle plate results with the recommended limits for microbial monitoring of clean areas shows that 97% of the settle plates from the BSC fulfilled the requirements for a grade A area (<1 CFU/4 h), and the rest of the settle plates from the BSC fulfilled the requirements for grade C (50 CFU/4 h) [20]. In addition, most of the settle plates (91%) from PR and MR fulfilled the recommendation for a grade C clean area, and only two settle plates exceeded grade D contamination (100 CFU/4 h).

### 3.3. Monitoring of Personnel after Compounding

According to the results obtained from finger dab plates, the least number of contaminated gloves was seen after compounding in the BSC (Table 3). However, only one participant managed to compound the products in the BSC without any contamination of the gloves in all three compounding situations. Much variation was present in the number of CFUs on gloves after compounding, with right- and left-hand gloves being contaminated or non-contaminated. Both gloves remaining non-contaminated was more often seen after compounding in the BSC than in the other two environments. Contamination of the left glove was more common than contamination of the right glove. None of the participants disinfected the gloves during compounding due to hospital guidelines.

Comparison of the results of finger dab plates (Table 3) with recommended limits shows that 54% of all finger dab plates fulfilled the requirements for grade A (<1 CFU/glove) and 91% for grade B (5 CFU/glove) areas [20]. Regarding the finger dab plates from the BSC, 67% fulfilled the requirements for a grade A clean area and 94% for a grade B clean area.

### 3.4. Sterility Test for Compounded Samples

Of the 220 samples, 5 were excluded from the results because both the compounded samples and the negative controls were contaminated. All excluded samples were filtrated simultaneously, which indicates that they were contaminated during sterility testing. 

Almost all (99%) of the compounded samples were sterile, and no significant differences were found in the contamination rates between the environments. One of the contaminated samples was compounded in the PR and filtrated after 24 h of storage. The other contaminated sample was compounded in the BSC and filtrated within 4 h of compounding. Participant 1, who compounded the contaminated sample in the PR, completed all steps included on the observation form. The finger dab plates were non-contaminated (Table 3) and the numbers of colonies on the settle plates were 11.4 CFU/4 h (TSA) and 24.8 CFU/4 h (SDA) (Table 2). Participant 3, who compounded the contaminated sample in the BSC, did not wear a protective gown, surgical face mask or hair cover. Participant 3 did not wash his/her hands before compounding and neglected to disinfect the septum of the vial before piercing it a second time. Nevertheless, the finger dab plates and settle plates taken during compounding were clean (BSC3, Table 2 and Table 3).

### 3.5. Contaminants

Both contaminated samples contained two phenotypically different colonies each (Supplementary Materials–Table S3). Contaminants were identified using the OmniLog ID System Protocol A, B and C1. The contaminants from the PR sample were identified as *Dietzia maris* and *Corynebacterium mycetoides*. Contaminants from the BSC sample were identified as *Paenibacillus castaneae* and *Staphylococcus capitis*.

## 4. Discussion

Even though aseptic techniques were followed relatively well in this study (Table 1), almost all (99%) of the compounding situations had at least one deficiency in aseptic technique. All participants disinfected their hands before compounding and wore non-sterile protective gloves. Washing hands was more frequent in some of the previous studies, while usage of non-sterile gloves and cleaning the compounding area were more frequent in this study [8,9,10,17,19]. It is noteworthy that earlier studies did not report whether participants disinfected their hands before compounding [8,9,10]. None of the participants used a surgical face mask and hair cover during compounding in the BSC. The lack of protective garments did not seem to affect the environment inside the cabinet. Hence, the hospital’s protocol of wearing a surgical face mask and hair cover while compounding in the BSC could be reviewed. Only one participant used a surgical face mask when compounding in the PR or MR, but this did not seem to affect the contamination rate of the compounded products. Use of proper protective garments is recommended especially when working in the PR and MR to minimize the risk of contamination [18]. Some additional training for aseptic techniques and the hospital’s compounding protocols could be recommended to improve the compliance and lower the risk of contamination.

The results of environmental monitoring varied markedly between participants (Table 2), which might have been partly a consequence of insufficient aseptic techniques. Contaminated settle plates from BSCs were taken during compounding by participants 10 and 12 (Table 2). Observations did not provide obvious reasons for the contaminated settle plate from participant 10. Participant 12 did not keep the airflow in the BSC on for 15 min prior to compounding, nor were packages of the equipment disinfected before placing them into the BSC, which might explain the contamination of the settle plate. Alternatively, rapid and careless working methods inside the BSC might be a cause for contamination, although these were not documented in this study. Short exposure time (5–31 min) of the settle plates can explain the high variation in the contamination rates since the low airborne microbial level requires a longer exposure time (e.g., 2–4 h) for precise results [23]. The compounding environment seemed to have a larger effect on the contamination rate of the settle plates than the aseptic technique used. Since hospital wards are not classified as clean areas, there are no specific guidelines for environmental monitoring. However, results show that the BSC is the most suitable environment for compounding of parenteral products in a clinical environment since the risk for microbial contamination from the surrounding is minimal. These results support the recommendation from Fimea to use the BSC whenever possible to prevent contamination [18]. Regarding finger dab plates, observations revealed that the higher contamination rate (≥10 CFU/glove) was commonly related to deficiencies in aseptic techniques such as insufficient disinfection of equipment, use of non-sterile gloves while preparing the equipment and cleaning of the compounding area.

The contamination rate for the compounded samples in this study is lower than the mean results of two systematic reviews (0.9% vs. 3.7% and 7.5%) [15,16]. Both reviews revealed varying contamination rates of the compounded samples in clinical environments (0.1–55.7%, *n* = 27 studies and 1.1–20.7%, *n* = 13 studies). 

No significant differences emerged in the contamination rates of the compounded samples between the three different environments. However, the results from the environmental monitoring (Table 2) showed that the BSC is the cleanest, and therefore the safest, compounding environment for preventing contamination of the compounded product. One of the contaminated samples was compounded in the PR. Even though aseptic techniques were followed and the finger dab plates were non-contaminated, the compounded sample and one of the settle plates were contaminated. The contaminant on the settle plate and the one in the contaminated sample were phenotypically similar, suggesting that air is a possible source of contamination. The other contaminated sample was from the BSC. In this case, both the settle plates and the finger dab plates were non-contaminated, but there were deficiencies in aseptic techniques. Non-contaminated settle plates from the BSC might be explained by the short exposure time (8 min). The source of contamination might be related to deviation from aseptic techniques such as not disinfecting the septum of the vial before piercing the septum a second time and not turning on the BSC for 15 min before beginning the compounding. 

The contaminated sample from the PR included Gram-positive bacteria (*Diezia maris* and *Corynebacterium mycetoides*). There are three case reports of infections, namely bacteraemia, aortitis and hip prosthesis infection, caused by *Diezia maris* [24,25,26]. No case reports of infections caused by *Corynebacterium mycetoides* were found. The other contaminated sample from the BSC included Gram-variable bacteria (*Paenibacillus castaneae*) and Gram-positive bacteria (*Staphylococcus capitis*) [27]. No case reports of *Paenibacillus castaneae* infections were found. However, several case reports described infections caused by *Staphylococcus capitis,* such as septicaemia and septic meningitis, even in preterm infants [28,29,30].

### Limitations of the Study

Participants in this study were volunteers, therefore, they might have been more interested in adhering to aseptic techniques than average workers. Participants could also have been working more carefully than usual since they were aware of the study and observation. Participants at ward 1 were not familiar with using the BSC prior to this study. Despite a training session on use of the BSC given by a clinical pharmacist, the lack of experience might have caused deficiencies in aseptic techniques, affecting the results. In addition, the compounding experience between the participants might be different and could affect the results. PR samples were compounded in empty rooms, which might have resulted in lower numbers of microbes on settle plates than in PR with patients present.

Although our results are similar to those of previous studies, we did not obtain significant differences in contamination rates of the compounded samples in the different environments. A larger number of compounded samples might have revealed significant differences in the contamination rates. In addition, the compounded samples might have been contaminated during membrane filtration. More similar studies are needed to verify these results.

## 5. Conclusions

To ensure patient safety in pediatric wards, high-quality compounding is necessary. Aseptic techniques were mostly sufficiently followed in all three study environments; however, there is room for improvement and additional training is recommended to improve the compliance. The compounding environment had a larger impact on microbial growth on the settle plates than the aseptic technique applied. The BSC is the most suitable environment for compounding of parenteral products in a clinical environment since the BSC fulfils the European Union Good Manufacturing Practices (EU GMP) guidelines for microbial monitoring to the greatest extent [20].

## Figures and Tables

**Table 1:** Results from the observations made during compounding of parenteral product samples in three different environments in hospital wards: patient room (PR, N = 36), medicine room (MR, N = 36) and biological safety cabinet (BSC, N = 36).

Aseptic Working Techniques Used While Compounding:	PR, Step Followed (%)	MR, Step Followed (%)	BSC, Step Followed (%)
Participant does not have infectious disease	100	100	100
Participant is not wearing jewellery or watches on his/her hands or wrists	100	100	100
Participant cleans the table with an alkaline cleanser	56	72	-
Participant cleans the table with 80% denaturized ethanol	78	75	-
BSC is kept on for 15 min before starting the compounding	-	-	92
Airflow of the BSC is at maximum speed	-	-	100
Participant washes hands with soap before compounding	44	56	67
Participant disinfects hands before compounding	100	100	100
Participant uses a disposable non-sterile protective gown	-	-	50
Participant uses a hair cover	-	-	0
Participant puts on a surgical face mask	11	11	0
Participant puts on non-sterile gloves before disinfecting the equipment to be used in the compounding and cleaning the compounding area	36	31	67
Participant cleans the cabinet with an alcoholic disinfectant	-	-	100
Participant places a sterile drape cover onto the benchtop in the BSC	-	-	50
Participant disinfects the equipment to be used in the compounding prior to use or when transferring it into the BSC	11	14	47
Participant disinfects his/her hands before changing the gloves	-	-	100
Participant changes the gloves before starting the compounding	100	100	100
Participant disinfects the septum of the vial	100	100	100
Front glass of the BSC is kept in working position	-	-	100
Participant opens sterile packages inside the BSC	-	-	92
Participant does not touch the connecting part of the syringe or the needle	100	100	97
Participant disinfects the septum of the vial before piercing it again	63 (*n* = 19)	20 (*n* = 14)	13 (*n* = 14)
Participant mixes the product by turning back and forth, not shaking	97	97	94
No spatters	-	-	69
Participant wipes possible spatters from the BSC immediately (if outside the drape cover)	-	-	0 (*n* = 11)
Participant cleans the BSC with an alcoholic disinfectant after compounding	-	-	50
Participant leaves the BSC on maximum or half speed after finishing the compounding and cleaning	-	-	83

N = number of compounding situations, N/A = not applicable, PR = patient room, MR = medicine room, BSC = biological safety cabinet, *n* = number of times step could have been followed (e.g., not every participant pierced the septum of a vial again).

**Table 2:** Microbial growth (CFU/4 h) on the settle plates, TSA (N = 108) and SDA (N = 108), during the compounding of parenteral product samples in the three different environments in hospital wards.

**Ward 1**	**Plate**	**PR1**	**PR2**	**PR3**	**MR1**	**MR2**	**MR3**	**BSC1**	**BSC2**	**BSC3**
Participant 1	TSA	24	11.4	24	42.4	14.1	0	0	0	0
	SDA	0	24.9	0	0	0	0	0	0	0
Participant 2	TSA	80	68.6	40	0	0	0	0	0	0
	SDA	0	34.3	80	0	0	0	0	0	0
Participant 3	TSA	60	0	30	180	26.7	0	0	0	0
	SDA	0	0	0	90	0	0	0	0	0
Participant 4	TSA	26.7	26.7	0	0	21.8	30	0	0	0
	SDA	26.7	0	0	0	0	0	0	0	0
Participant 5	TSA	0	0	0	0	0	0	0	0	0
	SDA	0	0	0	0	0	0	0	0	0
Participant 6	TSA	186.7	48	40	64.5	20	0	0	0	0
	SDA	53.3	0	0	0	20	0	0	0	0
Mean	TSA	37 CFU/4 h			22.25 CFU/4 h			0 CFU/4 h		
	SDA	12.17 CFU/4 h			6.11 CFU/4 h			0 CFU/4 h		
Combined	49.17 CFU/4 h				28.36 CFU/4 h			0 CFU/4 h		
**Ward 2**	**Plate**	**PR1**	**PR2**	**PR3**	**MR1**	**MR2**	**MR3**	**BSC1**	**BSC2**	**BSC3**
Participant 7	TSA	0	0	72	0	30	0	0	0	0
	SDA	0	0	0	0	0	0	0	0	0
Participant 8	TSA	0	0	13.3	30.9	17.1	0	0	0	0
	SDA	0	0	0	15.5	0	0	0	0	0
Participant 9	TSA	0	0	0	72	0	0	0	0	0
	SDA	0	0	0	0	48	40	0	0	0
Participant 10	TSA	0	30	0	0	0	0	0	0	0
	SDA	16	0	0	24	0	0	20	0	0
Participant 11	TSA	45.7 *	0	68.6	0	0	0	0	0	0
	SDA	11.4 *	0	0	40	0	0	0	0	0
Participant 12	TSA	34.3	48	96	0	0	68.6	21.8	0	0
	SDA	0	0	0	0	0	0	0	0	0
Mean	TSA	22.66 CFU/4 h			12.15 CFU/4 h			1.12 CFU/4 h		
	SDA	1.52 CFU/4 h			9.30 CFU/4 h			1.11 CFU/4 h		
Combined	24.18 CFU/4 h				21.45 CFU/4 h			2.23 CFU/4 h		

N = number of settle plates collected, PR = patient room, MR = medicine room, BSC = biological safety cabinet. Number after environment describes the order of the compounding situation: 1 = first compounding, 2 = second compounding, 3 = third compounding. Microbial growth on the settle plates taken during the compounding of contaminated product samples is written in bold. * Participant compounded four extra samples during this compounding situation.

**Table 3:** Results of the finger dab plates (N = 216). Results are presented as CFU/glove.

**Ward 1**	**Plate**	**PR1**	**PR2**	**PR3**	**MR1**	**MR2**	**MR3**	**BSC1**	**BSC2**	**BSC3**
Participant 1	Right	0	0	1	7	1	0	0	0	1
	Left	0	0	1	3	0	0	0	0	0
Participant 2	Right	0	0	1	0	0	0	0	1	0
	Left	1	3	0	0	0	1	7	0	1
Participant 3	Right	2	3	2	16	2	0	0	5	0
	Left	8	1	6	12	1	1	0	2	0
Participant 4	Right	0	0	1	1	0	3	2	0	0
	Left	1	2	2	3	0	2	0	0	0
Participant 5	Right	1	0	4	4	0	4	3	0	2
	Left	1	1	23	13	0	8	5	1	0
Participant 6	Right	1	0	0	0	1	5	0	2	0
	Left	2	1	2	5	3	1	0	2	0
Mean	Right	0.9			2.4			0.9		
	Left	3.1			2.9			1.0		
**Ward 2**	**Plate**	**PR1**	**PR2**	**PR3**	**MR1**	**MR2**	**MR3**	**BSC1**	**BSC2**	**BSC3**
Participant 7	Right	0	1	0	0	0	0	0	0	0
	Left	0	1	3	0	1	0	1	0	1
Participant 8	Right	0	0	4	13	0	3	1	0	0
	Left	0	0	1	0	2	2	0	2	0
Participant 9	Right	1	0	0	1	3	2	0	0	3
	Left	1	0	1	0	1	4	0	0	1
Participant 10	Right	0	0	0	6	9	4	1	1	2
	Left	0	1	1	6	10	0	2	0	0
Participant 11	Right	2 *	1	0	11	0	1	0	10	10
	Left	0 *	3	6	2	0	0	0	8	24
Participant 12	Right	1	0	0	0	0	0	0	0	0
	Left	4	1	1	0	1	1	0	0	0
Mean	Right	0.6			2.9			1.6		
	Left	1.3			1.7			2.2		

PR = patient room, MR = medicine room, BSC = biological safety cabinet. Number after environment describes the order of the compounding situation: 1 = first compounding, 2 = second compounding, 3 = third compounding. Finger dab plates related to compounding of contaminated product samples are written in bold. * Participant compounded four extra samples during this compounding situation.

## Data Availability

Data is contained within the article or supplementary material.

## Supplementary Materials

The following are available online at https://www.mdpi.com/article/10.3390/healthcare9081025/s1, Document S1: Instructions for compounding given to participants, Document S2: Observation forms, Table S3: Microbes identified by OmniLog ID System in the two contaminated product samples.

## References

[1] Council of Europe (2016). Parenteral preparations. European Pharmacopoeia.

[2] Staes C., Jacobs J., Mayer J., Allen J. (2013). Description of outbreaks of healt-care-associated infections related to compounding pharmacies, 2000–2012. Am. J. Health-Syst. Pharm..

[3] Habsah H., Zeehaida M., Van Rostenberghe H., Noraida R., Wan Pauzi W.I., Fatimah I., Rosliza A.R., Nik Sharimah N.Y., Maimunah H. (2005). An outbreak of *Pantoea* spp. in a neonatal intensive care unit secondary to contaminated parenteral nutrition. J. Hosp. Infect..

[4] Arslan U., Erayman I., Kirdar S., Yukekkaya S., Cimen O., Tuncer I., Bozdogan B. (2010). *Serratia marcescens* sepsis outbreak in neonatal intensive care unit. Pediatr. Int..

[5] Paul L., Hegde A., Pai T., Shetty S., Baliga S., Shenoy S. (2016). An outbreak of *Brunkholderia cepacia* Bacteremia in a Neonatal Intensive Care Unit. Indian J. Pediatr..

[6] Shrivastava B., Sriram A., Shetty S., Doshi R., Varior R. (2016). An unusual source of *Brunkholderia capacia* outbreak in a neonatal intensive care unit. J. Hosp. Infect..

[7] Breitkreutz B., Rane A., Schwab M., Hannsjörg M. (2011). Drug delivery and formulation. Pediatric Clinical Pharmacology.

[8] Cousins D., Sabatier B., Begue D., Schmitt C., Hoppe-Tichy T. (2005). Medication errors in intravenous drug preparation and administration: A multicentre audit in the UK, Germany and France. Qual. Saf. Health Care.

[9] Mendes J.R., Lopes M.C.B.T., Vancini-Campanharo C.R., Okuno M.F.P., Batista R.E.A. (2018). Types and frequency of errors in the preparation and administration of drugs. Einstein.

[10] Ong W., Subasyini S. (2013). Medication Errors in Intravenous Drug Preparation and Administration. Med. J. Malays..

[11] Suvikas-Peltonen E., Hakoinen S., Celikkayalar E., Laaksonen R., Airaksinen M. (2017). Incorrect aseptic techniques in medicine preparation and recommendations for safer practices: A systematic review. Eur. J. Hosp. Pharm..

[12] Turpin R., Solem C., Pontes-Arruda A., Sanon M., Mehta S., Xiaoqing Liu F., Botteman M. (2014). The impact of parenteral nutrition preparation on bloodstream infections risk and cost. Eur. J. Clin. Nutr..

[13] Thomas M., Sanborn M., Couldry R.I.V. (2005). Admixture contamination rates: Traditional practice site versus a class 1000 cleanroom. Am. J. Health Syst. Pharm..

[14] Stucki C., Sautter A.-M., Favet J., Bonnabry P. (2009). Microbial contamination of syringes during preparation: The direct influence of environmental cleanliness and risk manipulations on end-product quality. Am. J. Health-Syst. Pharm..

[15] Austin P.D., Hand K.S., Elia M. (2015). Systematic review and meta-analysis of the risk of microbial contamination of parenteral doses prepared under aseptic techniques in clinical and pharmaceutical environments: An update. J. Hosp. Infect..

[16] Larmené-Beld K., Frijlink H., Taxis K. (2019). A systematic review and meta-analysis of microbial contamination of parenteral medication prepared in a clinical versus pharmacy environment. Eur. J. Clin. Pharmacol..

[17] Austin P., Elia M. (2013). Improved aseptic technique can reduce variable contamination rates of ward-prepared parenteral doses. J. Hosp. Infect..

[18] (2012). Finnish Medicines Agency (Fimea): Lääkealan Turvallisuus ja Kehittämiskeskuksen Määräys 6/2012: Sairaala-Apteekin ja Lääkekeskuksen Toiminta. https://www.fimea.fi/documents/160140/764653/22690_Maarays_6_2012.pdf.

[19] Suvikas-Peltonen E., Palmgren J., Häggman V., Celikkayalar E., Manninen R., Airaksinen M. (2017). Auditing safety of compounding and reconstituting of intravenous medicines on hospital wards in Finland. Int. J. Pharm. Compd..

[20] (2008). EudraLex The Rules Governing Medicinal Products in the European Union Volume 4–EU Guidelines to Good Manufacturing Practice Medicinal Products for Human and Veterinary Use Annex 1: Manufacture of Sterile Medicinal Products (corrected version). European Comission: Brussels, Belgium. https://ec.europa.eu/health/sites/default/files/files/eudralex/vol-4/2008_11_25_gmp-an1_en.pdf.

[21] European Pharmacopoeia Commission (2016). European Pharmacopoeia: 5.1.9. Guidelines for using the test for sterility. European Pharmacopoeia.

[22] Sandle T., Skinner K., Sandle J., Gebala B., Kothandaraman P. (2013). Evaluation of the Gen III Omnilog^®^ ID System microbial identification system for the profiling of cleanroom bacteria. Eur. J. Pharm. Sci..

[23] A Working Group of the Scottish Quality Assurance Specialist Interest Group (2004). Guidelines on Test Methods for Environmental Monitoring for Aseptic Dispensing Facilities.

[24] Reyes G., Navarro J.-L., Gamallo C., dela Cuevas M.-C. (2006). Case report—Cardiac general: Type A aortic dissection associated with *Dietzia maris*. Interact. Cardiovasc. Thorac. Surg..

[25] Pidoux O., Argenson J.-N., Jacomo V., Drancourt M. (2001). Molecular identification of *Dietzia maris* hip prothesis Infection Isolate. J. Clin. Microbiol..

[26] Bemer-Melchior P., Haloun A., Riegel P., Drugeon H.B. (1999). Bacteremia due to *Dietzia maris* in an immunocompromised patient. Clin. Infect. Dis..

[27] Valverde A., Peix A., Rivas R., Velazquez E., Salazar S., Santa-Regina I., Rodriguez-Barrueco C., Igual J.M. (2008). *Paenibacillus castaneae* sp. nov., isolated from the phyllosphere of *Castanea sativa* Miller. Int. J. Syst. Evol. Microbiol..

[28] Ng P.C., Chow V.C.Y., Lee C.H., Ling J.M.L., Wong H.L., Chan R.C.Y. (2006). Persistent *Staphylococcus captis* septicemia in a preterm infant. Pediatr. Infect. Dis. J..

[29] Guen C.G.-L., Fournier S., Andre-Richet B., Caillon J., Espaze E., Richet H., Roze J.C., Lepelletier D. (2007). Almond oil implicated in a *Stephylococcus captis* outbreak in a neonatal intensive care unit. J. Perinatol..

[30] Wang S., Liu C., Tseng H., Yang Y.J., Lin C.H., Huang A.H., Wu Y.H. (1999). *Staphylococcus captis* basteremia of very low birth weight premature infants at neonatal intensive care units: Clinical significance and antimicrobial susceptibility. J. Microbil. Immunol. Infect..

