# Supplementary material for: Compounding Parenteral Products in Pediatric Wards—Effect of Environment and Aseptic Technique on Product Sterility
Source: Healthcare (Basel). 2021 Aug 10;9(8):1025. doi: 10.3390/healthcare9081025 (PMC8393854; doi:10.3390/healthcare9081025)
Supplement: Supplementary file 1 [file healthcare-09-01025-s001.zip › healthcare-1287953-supplementary.pdf]

healthcare

MDPI

Supplementary materials

Compounding parenteral products at pediatric wards – effect of environment and aseptic technique on product sterility

Sonja Virtanen [1], Karmen Kapp [2], Maria Rautamo [3], Lotta Schepel [3], Carita Lindén-Lahti [3], Cristina D. Cruz [2] and Päivi Tammela [2,*]

[1] Division of Pharmaceutical Biosciences, Faculty of Pharmacy, P.O.Box 56 (Viikinkaari 5E), FI-00014 University of Helsinki, Finland; sonja.a.virtanen@outlook.com
[2] Drug Research Program, Division of Pharmaceutical Biosciences, Faculty of Pharmacy, P.O.Box 56 (Viikinkaari 5E), FI-00014 University of Helsinki, Finland; karmen.kapp@helsinki.fi (K.K); cristina.durantecruz@helsinki.fi (C.D.C.); paivi.tammela@helsinki.fi (P.T.)
[3] HUS Pharmacy, HUS Helsinki University of Hospital and University of Helsinki, Stenbäckinkatu 9B, 00290 Helsinki, Finland; maria.rautamo@hus.fi (M.R.); lotta.schepel@hus.fi (L.S.); carita.linden-lahti@hus.fi (C.L.-L.)
* Correspondence: paivi.tammela@helsinki.fi; Tel.: +358-50-4480886

Document S1: Instruction for compounding given to participants.

Instruction for sample compounding

Compounding should be done in the same way as normally during the workday. Additionally, settle plates will be opened on the table or in the biological safety cabinet before starting the compounding. After compounding, the participant will give finger dab plates to the agar plate.

1. Open the settle plates on the table or in the biological safety cabinet.
2. Start compounding.
3. Add 5 mL of sterile water for the injections through mini-spike to the injection bottle.
4. Let the powder dissolve.
5. Take 2 mL of solution through mini-spike to two syringes (10 mL).
6. Dilute the solution by adding sterile NaCl solution (0.9%) to the syringes (ad 10 mL) with another syringe and needle.
7. Close the solution syringe with a sterile cap.
8. Give finger dab plates.

Document S2: Observation forms.

Compounding in patient room:	Participant 1	Participant 2
Participant does not have infectious disease		
Participant is not wearing jewellery or watches on his/her hands or wrists		
Participant cleans the table with an alkaline cleanser		
Participant cleans the table with 80% denaturized ethanol		
Participant washes hands with soap before compounding		
Participant disinfects hands before compounding		
Participant uses a disposable non-sterile protective gown		
Participant uses a hair cover		
Participant puts on a surgical face mask		

Participant puts on non-sterile gloves before disinfecting the equipment to be used in the compounding and cleaning the compounding area		
Participant disinfects his/her hands before changing the gloves		
Participant changes the gloves before starting the medicine compounding		
Participant disinfects the septum of the vial even though it has manufacturer's cap on		
Participant does not touch the connecting part of the syringe or the needle		
Participant disinfects the septum of the vial before piercing it again		
Participant mixes the product by turning back and forth, not shaking		

Compounding in medicine room:	Participant 1	Participant 2
Participant does not have infectious disease		
Participant is not wearing jewellery or watches on his/her hands or wrists		
Participant cleans the table with an alkaline cleanser		
Participant cleans the table with 80% denaturized ethanol		
Participant washes hands with soap before compounding		
Participant disinfects hands before compounding		
Participant uses a disposable non-sterile protective gown		
Participant uses a hair cover		
Participant puts on a surgical face mask		
Participant puts on non-sterile gloves before disinfecting the equipment to be used in the compounding and cleaning the compounding area		
Participant disinfects his/her hands before changing the gloves		
Participant changes the gloves before starting the medicine compounding		
Participant disinfects the septum of the vial even though it has manufacturer's cap on		
Participant does not touch the connecting part of the syringe or the needle		
Participant disinfects the septum of the vial before piercing it again		
Participant mixes the product by turning back and forth, not shaking		

Compounding in laminar biological safety cabinet:	Participant 1	Participant 2
Participant does not have infectious disease		
Participant is not wearing jewellery or watches on his/her hands or wrists		
Participant cleans the table with an alkaline cleanser		
Participant cleans the table with 80% denaturized ethanol		
The BSC is turned on for 15 minutes before starting the compounding		
Airflow of the BSC is at maximum speed		
Participant washes hands with soap before compounding		
Participant disinfects hands before compounding		
Participant uses a disposable non-sterile protective gown		
Participant uses a hair cover		
Participant puts on a surgical face mask		
Participant puts on non-sterile gloves before disinfecting the equipment to be used in the compounding and cleaning the compounding area		
Participant cleans the cabinet with an alcoholic disinfectant		
Participant places a sterile drape cover onto the benchtop in the BSC		
Participant disinfects the equipment to be used in the compounding before the compounding or putting them into the BSC		
Participant disinfects his/her hands before changing the gloves		

Participant changes the gloves before starting the medicine compounding
Participant disinfects the septum of the vial even though it has manufacturer's cap on
Front glass of the BSC is kept in working position
Participant opens sterile packages inside the BSC
Participant does not touch the connecting part of the syringe or the needle
Participant disinfects the septum of the vial before piercing it again
Participant mixes the product by turning back and forth, not shaking
No spatters

Table S3. Microbes identified by OmniLog ID System in the two contaminated product samples.

Compounding area	Phenotype of the CFU	Number of CFU/ml	Identified contaminant	Omnilog ID System protocol used
PR	Bright orange, big, round convex, shiny	1.89×10^6	*Dietzia maris*	Protocol A
PR	Bright yellow, small, round, convex, shiny	0.38×10^6	*Corynebacterium mycetoides*	Protocol A
BSC	Light yellow, big, round, flat, shiny	1.8×10^4	*Paenibacillus castanea*	Protocol B
BSC	Light yellow (almost white), small, round, flat, shiny	0.21×10^6	*Staphylococcus capitis*	Protocols A and C1
